# FlgI Is a Sec-Dependent Effector of *Candidatus* Liberibacter asiaticus That Can Be Blocked by Small Molecules Identified Using a Yeast Screen

**DOI:** 10.3390/plants13020318

**Published:** 2024-01-21

**Authors:** Siliang Zuo, Linghui Xu, Huiyan Zhang, Meiqian Jiang, Sifeng Wu, Lian-Hui Zhang, Xiaofan Zhou, Junxia Wang

**Affiliations:** Integrative Microbiology Research Centre, Guangdong Province Key Laboratory of Microbial Signals and Disease Control, South China Agricultural University, Guangzhou 510642, China; slzuo0731@163.com (S.Z.); xulinghui@ymail.com (L.X.); 15218121539@163.com (H.Z.); jiangmi3275@163.com (M.J.); emmcfong@163.com (S.W.); lhzhang01@scau.edu.cn (L.-H.Z.)

**Keywords:** citrus huanglongbing, *Candidatus* Liberibacter asiaticus, sec-dependent effector, *Saccharomyces cerevisiae*, *Nicotiana benthamiana*, small molecule inhibitors

## Abstract

Huanglongbing (HLB) is one of the most devastating diseases of citrus worldwide. The phloem-restricted bacterium *Candidatus* Liberibacter asiaticus (CLas) is considered to be the main pathogen responsible for HLB. There is currently no effective practical strategy for the control of HLB. Our understanding of how pathogens cause HLB is limited because CLas has not been artificially cultured. In this study, 15 potential virulence factors were predicted from the proteome of CLas through DeepVF and PHI-base searches. One among them, FlgI, was found to inhibit yeast growth when expressed in *Saccharomyces cerevisiae*. The expression of the signal peptide of FlgI fused with PhoA in *Escherichia coli* resulted in the discovery that FlgI was a novel Sec-dependent secretory protein. We further found that the carboxyl-terminal HA-tagged FlgI was secreted via outer membrane vesicles in *Sinorhizobium meliloti*. Fluoresence localization of transient expression FlgI-GFP in *Nicotiana benthamiana* revealed that FlgI is mainly localized in the cytoplasm, cell periphery, and nuclear periphery of tobacco cells. In addition, our experimental results suggest that FlgI has a strong ability to induce callose deposition and cell necrosis in *N. benthamiana*. Finally, by screening a large library of compounds in a high-throughput format, we found that cyclosporin A restored the growth of FlgI-expressing yeast. These results confirm that FlgI is a novel Sec-dependent effector, enriching our understanding of CLas pathogenicity and helping to develop new and more effective strategies to manage HLB.

## 1. Introduction

Huanglongbing (HLB) is one of the most serious citrus diseases worldwide [1,2,3,4,5]. Its typical symptoms include the yellowing of the leaf veins, wilting of branches, and recession of roots, ultimately leading to the death of the affected trees [1,3]. Based on geographic distribution and the 16S rDNA sequence, HLB is associated with a phloem-restricted bacterium *Candidatus* Liberibacter asiaticus (CLas), *Ca*. L. africanus, or *Ca*. L. americanus. CLas is the most prevalent pathogen with global distribution [1]. From 2007 to 2018, CLas led to a significant reduction in the orange juice and fresh fruit market in the United States [6]. In southern China’s citrus-growing regions, tens of millions of diseased trees have been destroyed in the past few years [7]. All commercial citrus cultivars and several citrus relatives are susceptible to CLas, for which there is currently no cure for HLB [3,8]. Therefore, there is an urgent need for the effective management of this disease in the market. However, the lack of understanding of CLas virulence mechanisms has hindered the development of effective HLB management.

CLas has not been cultured in vitro; however, the availability of CLas genomic information [9,10] has contributed to the research of its virulence mechanism. Bacterial pathogens often secret proteins (called effectors) that are involved in disease pathogenesis [11]. Unlike other Gram-negative bacteria that have evolved type III, IV, or VI secretion systems to deliver effectors directly to host cells [12,13,14], CLas lacks these systems; despite this, it has a general secretory pathway [9] and secretes at least 86 proteins [15]. Several reports have been published on the use of transient expression to identify CLas effectors [8,16,17,18,19]. Las5315, also called Sec-delivered effector 1 (SDE1), induced callose deposition, cell death, and leaf chlorosis in infiltrated *Nicotiana benthamiana* leaves through transient expression [8,20] and inhibited the activity of citrus papain-like cysteine proteases to suppress citrus defense responses [16]. The expressed NLS-m460 containing an SV40 nuclear localization sequence in the N-terminus through the Potato virus X -based expression vector in *N. benthamiana* caused local leaf chlorosis and the severe necrosis of systemic leaves [17]. Overexpressing mature secretion proteins m3875 and m4405 inhibited programmed cell death and hydrogen peroxide accumulation induced by BAX and INF1. Overexpression also led to stunting and leaf deformation phenotypes, suggesting that m3875 and m4405 have roles in plant immune response, growth, and development [19,21]. The overexpression of CLIBASIA_00470 and CLIBASIA_04025 significantly inhibits the growth of *N. benthamiana* at 15 days post infiltration [18]. In addition, the CLIBASIA_04025 was found to target CsACD2 to promote a devastating HLB disease [22]. At 5 weeks after infiltration, the phloem necrosis of the senescent leaves of *N. benthamiana* with heterologous expression of m03915 and m04250 was observed [23]. The effector CLas4425 contributes to virulence in CLas, probably by interfering with SA-mediated plant immunity [24]. A few of the SDEs were expressed at higher levels in citrus than they were in psyllid [15,24,25,26], indicating that they may contribute to CLas colonization and disease development in citrus. However, little is known about the cellular functions of CLas SDEs in plant hosts. Therefore, exploring the roles of novel CLas SDEs or virulence factors (VFs) will help in understanding the pathogenic mechanisms of CLas and disease control.

Budding yeast has become a useful tool for discovering the function of bacterial effectors [27,28,29]. This strategy was designed based on the fact that bacterial effectors target conserved cell machinery between yeast and other eukaryotes [28,30]. The heterologous expression of the effectors of many plant and animal pathogens leads to robust phenotypes such as growth inhibition. A number of effectors have been previously identified using this method, including HopAA1-1, HopAD1, HopU1, HopX1, HopG1, and AvrPtoB of *Pseudomonas syringae* pv. *tomato* [31,32]; XopB, XopE1, XopF2, XopX, and AvrRxo1 of *Xanthomonas campestris* pv. *vesicatoria* [29]; and ExoA, ExoS, ExoT, and ExoY of *Pseudomonas aeruginosa* [33,34,35,36]. Yeast cell-based assays are also used for drug discovery, usually through large-scale screening for molecules that restore the yeast cell growth inhibited by toxins or proteases [36,37,38].

In this study, 15 VFs were predicted from the proteome of CLas using DeepVF and PHI-base searches in preparation for further experimental studies. By exploiting heterologous expression in *Saccharomyces cerevisiae*, one of them, the flagellar basal body P-ring protein FlgI (CLIBASIA_01305), was found to inhibit yeast growth. An *Escherichia coli* alkaline phosphatase (*PhoA*) gene fusion assay and outer membrane vesicles protein identification confirmed that FlgI is a novel Sec-dependent secretory protein. The localization and biological function of FlgI were determined through the transient expression of FlgI in *N. benthamiana* leaves. In addition, large-scale inhibitors were used to screen in yeast cell-based assays, discovering new molecule inhibitors that can restore the proliferation of FlgI-expressing yeast cells. These results deepen our understanding of CLas pathogenesis and contribute to the development of new and more effective treatment strategies for HLB.

## 2. Results

### 2.1. Fifteen Putative Effector Proteins of CLas Were Identified by Informatics Analysis

In a previous study, Pitino et al. [8] predicted 16 candidate VFs of CLas based on multiple sequence features, including the presence of signal peptide, lack of transmembrane domain, short protein length, and lack of known functions. The functional investigation of these putative effectors led to the identification of SDE1. To expand our knowledge of the pathogenicity of CLas, we set out to screen for additional CLas effectors. To this end, we analyzed the predicted proteome of CLas using DeepVF, a deep learning-based computational pipeline that utilizes an ensemble of machine learning algorithms and features to achieve high effectiveness in predicting bacterial VFs. DeepVF predicted 780 potential CLas VFs with a default prediction score threshold of 0.5 and 268 putative VFs with a more stringent threshold of 0.85 (Supplementary Table S1). In addition, we compared the predicted proteome of CLas against the PHI-base database and identified 309 proteins with significant matches to known VFs (Supplementary Table S1). The large number of predicted VFs might suggest that many proteins are involved in the pathogenicity of CLas; at the same time, the results of the genome-scale bioinformatic prediction likely contain considerable false positives. Therefore, we randomly selected 15 predicted VFs predicted using DeepVF (with various prediction scores) and/or PHI-base searches for further experimental investigation (Table 1).

### 2.2. FlgI Was Identified as a Potential CLas Virulence Factor in a Yeast Cell-Based System

**Figure 1:**
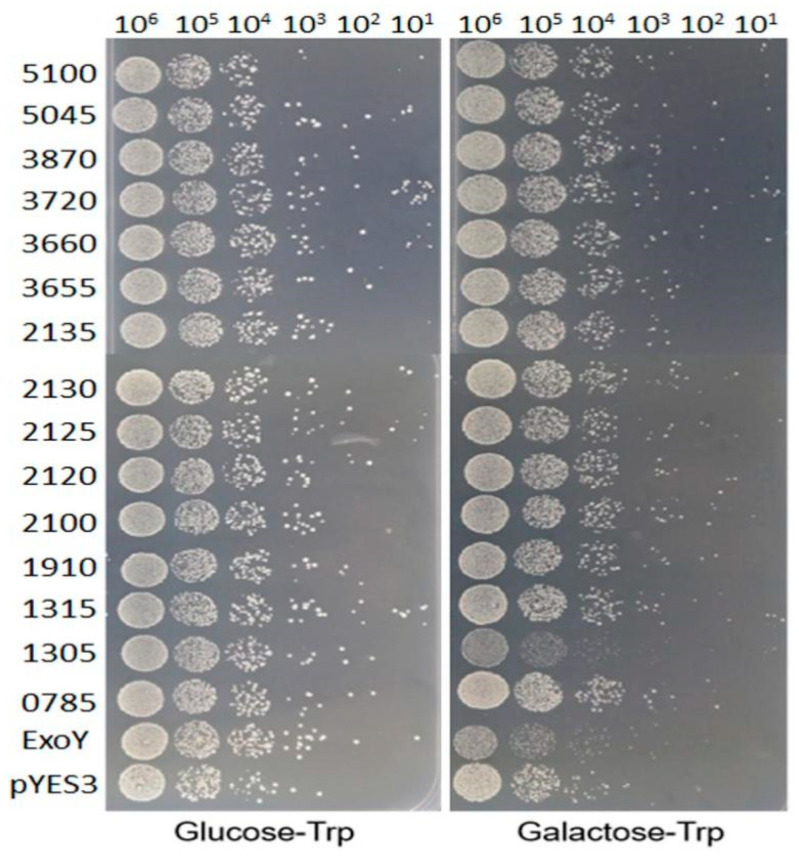
Yeast growth was inhibited by the heterologous expression of a CLas protein FlgI (CLIBASIA_01305). The 15 potential virulence factors of CLas were individually transferred into the yeast expression vector pYES3/CT. Transformed yeast was grown overnight in the repressing medium (2% glucose). Cultures were then normalized to OD_600_ of 1.0, and serial 10-fold dilutions were spotted onto a repressing or inducing solid medium (2% galactose and 1% raffinose). Cell growth was compared to the yeast with the empty vector (pYES) and to the yeast harboring the toxic gene Exoenzyme Y of *P. aeruginosa* (positive control). Photographs were taken after 2 to 3 days of growth at 30 °C. The experiment was performed three times.

CLas, the causal bacterium of HLB disease, has yet to be cultured. To elucidate the function of effectors, budding yeast *S. cerevisiae* was used as a surrogate host [27,28,29]. Here, we performed yeast cell-based assays to determine whether any of the 15 predicted VFs described above had the ability to inhibit the growth of yeast cells when heterologously expressed in *S. cerevisiae* strain W303-1A. These 15 VFs were constructed into the yeast expression vector pYES3/CT under the control of the GAL1 promoter, respectively. The *S. cerevisiae* cells carrying pYES3/CT-derived plasmids were cultured, and 2 μL aliquots of 10 × serial dilution samples were spotted on agar plates containing either 2% galactose + 1% raffinose (the expression of these effectors was induced) or 2% glucose (as controls, the expression of effectors was not induced). We observed that only the colonies carrying the FlgI (CLIBASIA_01305) impaired the growth of yeast (Figure 1). These results indicate that FlgI is a potential effector inhibiting yeast cell growth.

### 2.3. Expression of FlgI Causes a Reduction in Yeast Cell Proliferation

**Figure 2:**
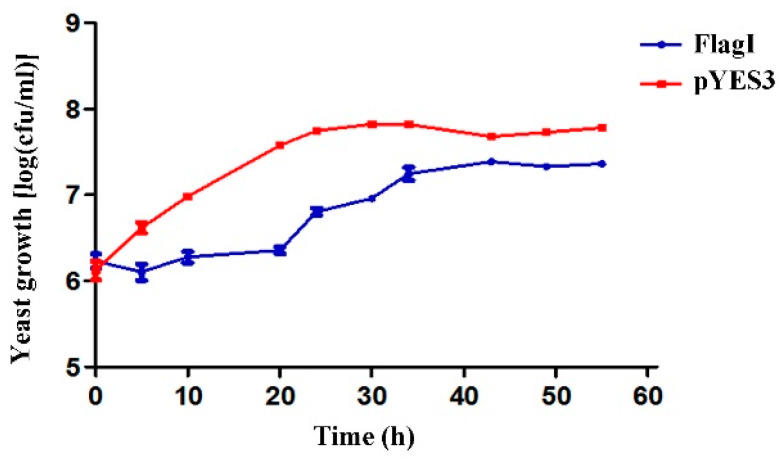
Cell viability assays of yeast strain expressing FlgI, which inhibits yeast growth. A W303-1A yeast strain carrying plasmid for the expression of FlgI, or containing an empty vector pYES3, was grown overnight in a repressing medium. Cultures were diluted to OD_600_ of 0.5 and allowed to recover in a fresh repressing medium for 2 h prior to washes, normalization to OD_600_ of 0.2, and incubation in inducing medium. For each time point, an aliquot was removed from the cultures, and serial 10-fold dilutions were spotted onto the repressing medium to assess the number of viable cells. The assay was performed three times with similar results. Bars represent the mean ± standard error.

To determine whether the growth inhibition of yeast cells caused by the high-level expression of FlgI was a result of cytotoxicity or the arrest of cell growth, cell viability assays were performed with plasmid-carrying yeast strains grown in a medium with an induced expression of FlgI. Yeast cells expressing FlgI were collected at indicated time points and the 10-fold serially diluted samples were spread onto the surface of the repressing solid medium. The number of viable cells present on each plate was counted. The results showed a significantly decreased number of viable cells over time for the yeast strain carrying the FlgI plasmid compared to that of the empty vector control (Figure 2). These results suggest that the expression of FlgI in yeast leads to a reduction in cell proliferation.

### 2.4. FlgI Was a Sec-Dependent Presecretory Protein

**Figure 3:**
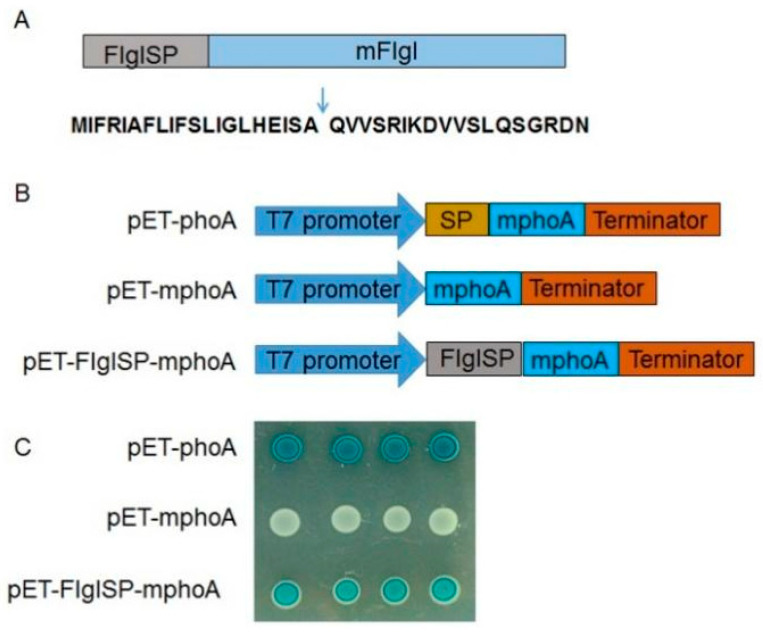
FlgI is a Sec-dependent presecretory protein: (**A**) Primary structure of FlgI. The arrow indicates the predicted cleavage site. The putative signal peptide (SP) of FlgI was designated FlgISP, and the mature form of FlgI was designated mFlgI. (**B**) Schematic of the prokaryotic expression cassettes for the alkaline phosphatase (*phoA*) gene. pET-phoA harboring the full-length *phoA* gene was used as a positive control, while pET-mphoA was a negative control that harbors the mature phoA lacking its native SP-encoding sequence. (**C**) FlgISP directed the extracellular translocation of the mPhoA moiety. After 16 h of incubation at 37 °C on LB media containing BCIP (90 μg/mL), IPTG (100 mM) and Na_2_HPO_4_ (75 mM), *E. coli* cells expressing the fusion protein FlgISP-mphoA turned blue.

The signal peptide (SP) of FlgI was predicted using SignalP 5.0, Phobius, and SPOCTOPUS. The FlgI cleavage site is located in 20–21 aa (Figure 3A). To verify the export of FlgI through the Sec translocon, an *Escherichia coli* (*E. coli*) *phoA* gene fusion analysis was performed as in previous reports [15,17]. On indicator LB agar with 5-bromo-4-chloro-3-indolylphosphate (BCIP) and Na2HPO4, *E. coli* cells containing pET-mphoA remained white after 16 h incubation; however, cells haboring pET-FlgISP-mphoA turned dark blue over 16 h of incubation (Figure 3B,C), which indicates that FlgISP directed the extracytoplasmic translocation of the mPhoA moiety. Taken together, the signal peptide prediction combined with the above experimental data suggest that FlgI represents a typical Sec-dependent secretory protein, which is consistent with the previous report [15].

### 2.5. FlgI Was Secreted via Outer Membrane Vesicles in Sinorhizobium meliloti

**Figure 4:**
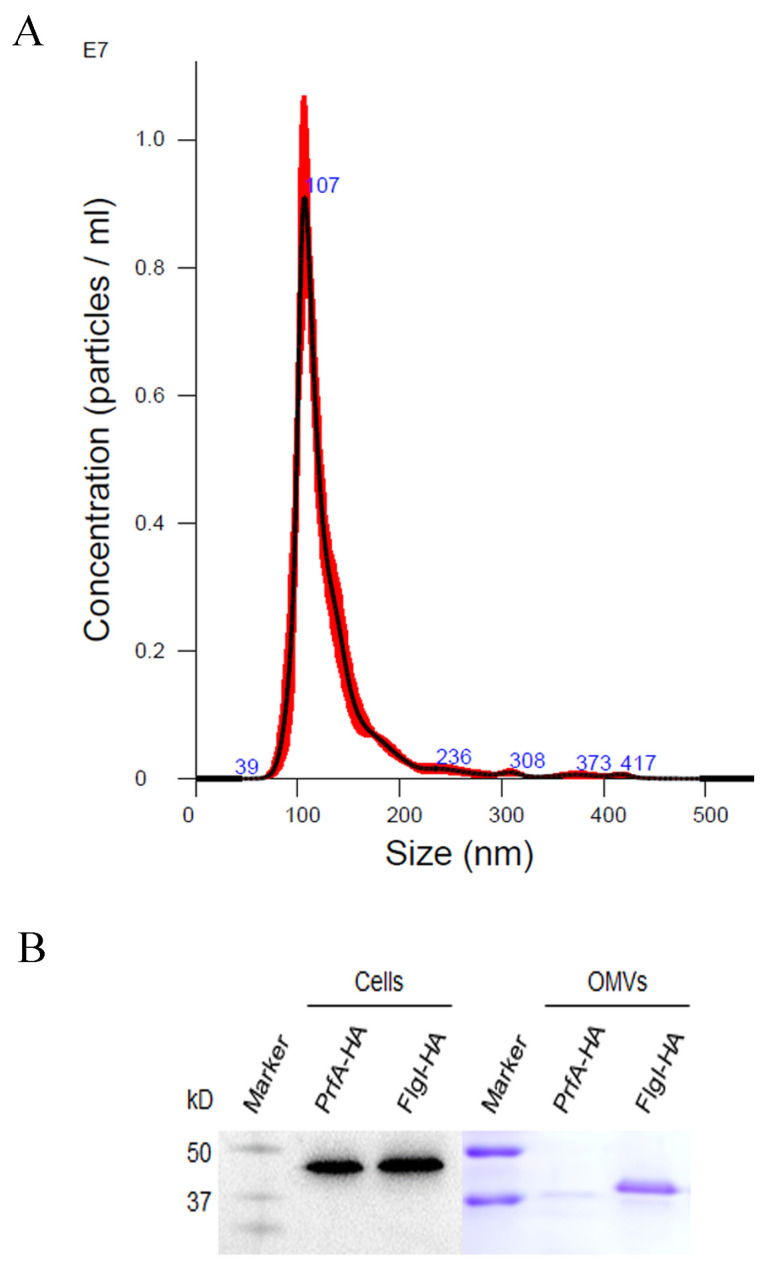
FlgI was secreted via outer membrane vesicles in *Sinorhizobium meliloti*: (**A**) The size distribution and amount of outer membrane vesicles in *S. meliloti* were analyzed using NanoSight LM10 nanoparticle tracking analysis (NTA 3.0). The red error bar indicates + / −1 standard error of the mean. The blue number indicates the size of the peak (nm). (**B**) Protein concentrations were determined using a Bradford assay. Purified proteins in cells or OMVs were separated in 10% SDS polyacrylamide gels and then detected using Western blot analysis or stained with Coomassie brilliant blue. The PrfA of *Xanthomonas citri* subsp. *citri*, which has a similar molecular weight to FlgI, was used as a control.

*Liberibacter crescens* can deliver proteins to the extracellular compartment via outer membrane vesicles (OMVs) [39], and this has been shown to secrete various VFs in other pathogens [40]. Because CLas is yet to be cultivated, the closely related model bacterium, *S. meliloti* [9], was used as a surrogate to identify proteins in the OMVs. We expressed carboxyl-terminal, HA-tagged FlgI in *S. meliloti* and purified OMVs from cell culture media using Amicon ultra-15 centrifugal filters and size-exclusion chromatography as previously described [41]. The sizes of the OMVs (diameter) released from *S. meliloti* were predominantly 107 nm, ranging from 30 nm to 400 nm (Figure 4A). The proteins of OMVs were extracted and analyzed using SDS-PAGE. The OMV sample had a significant band at the molecular weight corresponding to FlgI-HA, where we detected the band using Western blots (Figure 4B). These results represent the first experimental evidence that CLas can deliver the virulence factor FlgI via OMVs to the extracellular compartment.

### 2.6. The Subcellular Localization of FlgI in N. benthamiana

**Figure 5:**
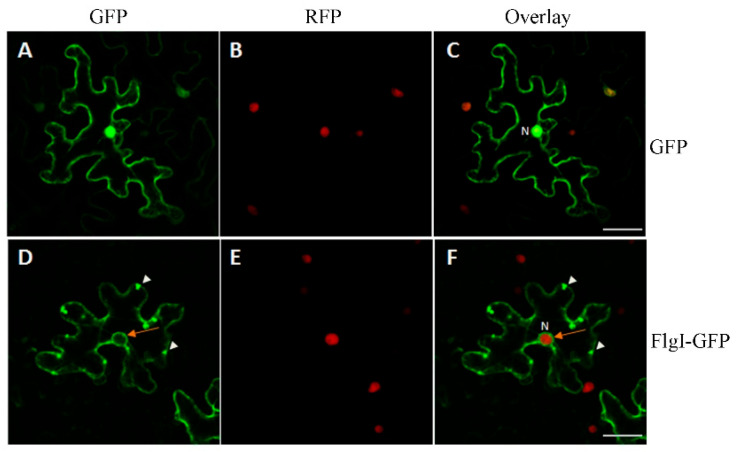
Subcellular localization of FlgI in *Nicotiana benthamiana.* pC35S-FlgI-GFP and pC35S-GFP were individually delivered into the leaves of the five-leaf stage *N. benthamiana* plants grown at 25 °C via agroinfiltration. The fluorescence was viewed using a Nikon A1 confocal microscope after 48 h post-inoculation. (**A**–**C**) Images show localization of GFP control in *N. benthamiana*; (**D**–**F**) Images show localization of FlgI-GFP *N. benthamiana*. The left panel shows GFP fluorescence (green), the middle panel shows the RFP fluorescence of the red nuclear marker RFP–H2B protein, and the right panel shows the overlay of GFP and RFP. The orange arrow indicates the nuclear periphery, and the white arrowheads show strong speckle fluorescence signals along the cytoplasmic periphery. N, nucleus. Scale bars represent 5 μm.

Since CLas is an intracellular pathogen, FlgI can be released directly into host cells. To facilitate the subcellular localization of FlgI in *N. benthamiana* cells, we constructed the vector pC35S-FlgI-GFP to express the FlgI fused with the GFP tag at its carboxyl-terminal end and transformed it into *N. benthamiana* RFP–H2B leaf cells for transient expression [42]. The cell localization of FlgI-GFP under a Nikon A1 confocal microscope (Nikon Imaging Japan Inc., Tokyo, Japan) showed that it was mainly distributed in the cytoplasm, cell periphery, and nuclear periphery (indicated by the orange arrow), with strong speckle fluorescence signals along the cell periphery (indicated by white arrowheads) (Figure 5). In contrast, the GFP control was dispersed in the cytoplasm, cell periphery, and nucleus, where it overlapped with the red nuclear marker RFP–H2B protein in the nucleus (Figure 5).

### 2.7. FlgI Induced Cell Death, Electrolyte Leakage, and Callose Deposition in N. benthamiana

**Figure 6:**
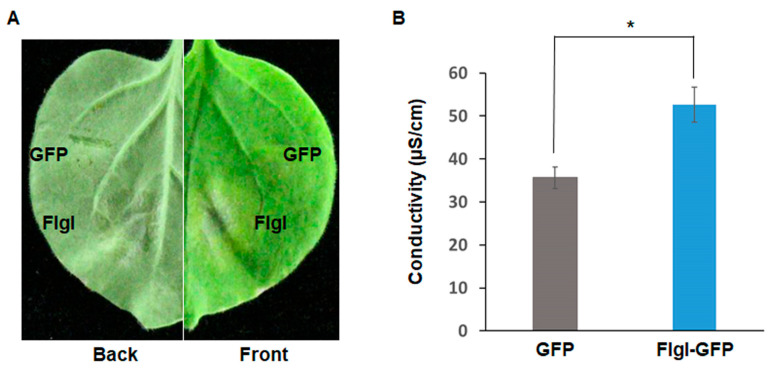
Cell death and electrolyte leakage in *N. benthamiana* leaves induced by transient expression of FlgI: (**A**) The *Agrobacterium* cells containing the pC35S-FlgI-GFP or pC35S-GFP were injected into the leaves of *N. benthamiana*. The plants were cultivated in a constant temperature plant incubator at 26 °C. Photographs were taken 7 days post infiltration (dpi). The left panel shows the abaxial side of the leaf; the right panel shows the adaxial side. The experiments were performed in 3 independent replicates. (**B**) Electrolyte leakage induced by FlgI-GFP was measured in the infiltrated leaves at 7 dpi. Mean values were analyzed and separated using Tukey’s HSD test at * *p* < 0.05 after one-way ANOVA. Error bars represent standard deviation.

To further define whether the FlgI effector induces a hypersensitive reaction in plants, the leaves of tobacco plants were inoculated with *Agrobacterium tumefaciens* (*A. tumefaciens*) carrying plasmid pC35S-FlgI-GFP or pC35S-GFP. The results showed that the tobacco leaves infiltrated with *Agrobacterium* cells carrying plasmid pC35S-FlgI-GFP developed clear lesions reminiscent of the hypersensitive response at 7 days post infiltration (dpi). In contrast, the negative control leaves infiltrated with pC35S-GFP did not have any necrotic spots (Figure 6A). These results indicate that the heterologous expression of FlgI induces cell death and a hypersensitive reaction in tobacco. In addition, an electrolyte leakage assay was employed to quantify the cell death induced by pC35S-FlgI-GFP. The results showed that significant amounts of ion leakage were detected, namely, 7 dpi with the *Agrobacterium* cells carrying the pC35S-FlgI-GFP (Figure 6B).

**Figure 7:**
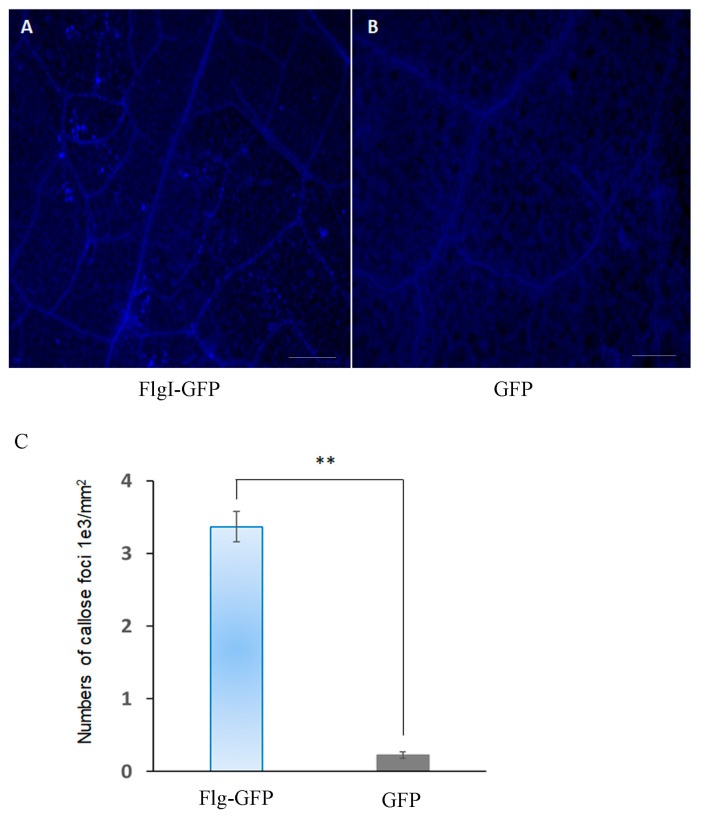
Callose deposition induced by the transient expression of FlgI in *N. benthamiana*: (**A**,**B**) The *Agrobacterium* cells containing the pC35S-FlgI-GFP or pC35S-GFP were injected into the leaves of *N. benthamiana*, and the plants were cultivated at 26 °C. Leaves were collected at 3 dpi and then stained with aniline blue. Callose deposition was observed under a UV epifluorescence microscope. Scale bars represent 200 μm. (**C**) Relative callose deposition was measured in the infiltrated leaves at 3 dpi. The number of foci of callose deposition counted in per mm^2^. Mean values were analyzed and separated using Tukey’s HSD test at ** *p* < 0.01 after one-way ANOVA. Error bars represent standard deviation.

Plants usually initiate an immune response to defend themselves in response to environmental stresses or pathogen infection. Callose is a carbohydrate that accumulates around the edge of the plant sieve. It has the physiological characteristics of rapid polymerization and depolymerization. Its accumulation can enhance the strength of the cell wall and help prevent the invasion of phytopathogens [43]. Compared with the pC35S-GFP vector control, fluorescence microscope aniline blue staining showed that pC35S-FlgI-GFP induced stronger callose deposition in the infiltration area of tobacco leaves 3 dpi (Figure 7).

### 2.8. Transiently Expressed FlgI Enhanced the Expression of Genes Related to Plant Defense in N. benthamiana

**Figure 8:**
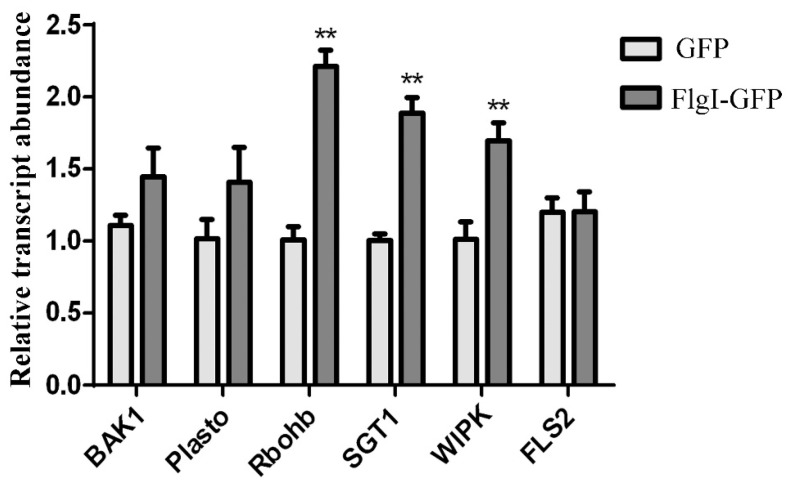
Transient expression of FlgI induced changes in the expression of defense-associated genes in *N. benthamiana*. *N. benthamiana* leaves were agroinfiltrated with the pC35S-FlgI-GFP or pC35S-GFP. The infiltrated zones were harvested at 2 dpi for RNA isolation. RT-qPCR was performed to measure the relative expression levels of *NbBAK1*, *NbFLS2*, *NbSGT1*, *NbPlastocyanin*, *NbRbohB*, and *NbWIPK*. The samples were normalized against *NbEF1α*. The data show the average folded gene induction in response to vector-infiltrated samples from three independent experiments. Bars represent the mean ± standard error, and asterisks signs indicate statistically significant differences from the control as calculated using Student’s *t*-test (** *p* < 0.01).

The pathogen infection-triggered immunity response resulted in the upregulation of many defense genes, including genes involved in pathogen-associated molecular patterns (PAMPs) [42,43,44]. Because FlgI induced a hypersensitive reaction in tobacco, we next detected the transcription level of the PAMP-associated genes in the *N. benthamiana* leaves containing FlgI. The total RNA was extracted for the tobacco leaves’ infiltration with *A. tumefaciens* containing FlgI-GFP or pC35S-GFP plasmid and then subjected to a reverse transcriptase quantitative PCR (RT-qPCR) reaction. The highly conserved N-terminal domain of flagellin is a plant bacterial PAMP. Bacterial flagellin is recognized by the leucine-rich repeating protein kinase-like receptor flagellin sensitive 2 (FLS2), which interacts with BAK1 and SGT1 [45,46]. As a result, we discovered that the expression levels of *NbFLS2* and *NbBAK1* showed no obvious differences 2 dpi; meanwhile, *NbSGT1* expression was elevated 1.9-fold 2 dpi in the *N. benthamiana* leaves that transiently expressed FlgI compared with those that expressed GFP (Figure 8). In *N. benthamiana*, respiratory burst oxidase (*RbohB*), a homolog of *RbohD*, is essential for the production of reactive oxygen species (ROS), while silencing *NbRbohB* can completely eliminate ROS bursts [47,48]. *NbRbohB* expression was upregulated 2.2-fold 2 dpi in the *N. benthamiana* leaves that transiently expressed FlgI compared with those that expressed GFP (Figure 8). In *N. benthamiana,* wound-induced protein kinase (WIPK) is activated quickly after elicitation [47]. Furthermore, WIPK is essential for bacterial immunity in *N. benthamiana* [43,47]. *NbWIPK* increased 1.7-fold 2 dpi in the *N. benthamiana* leaves that transiently expressed FlgI compared with those that expressed GFP (Figure 8). Plastocyanin plays a key role in photosynthesis and has been reported to be induced in a PAMP-triggered immune response to non-pathogenic *Pseudomonas fluorescens* [49]. In our experiment, the transcript abundance of *NbPlastocyanin* showed no obvious differences 2 dpi in the *N. benthamiana* leaves that transiently expressed FlgI compared with those that expressed GFP (Figure 8). Taken together, our results suggest that FlgI induces the expression of PAMP-triggered genes in *N. benthamiana*.

### 2.9. Identification of Small Molecular Inhibitors of FlgI

**Figure 9:**
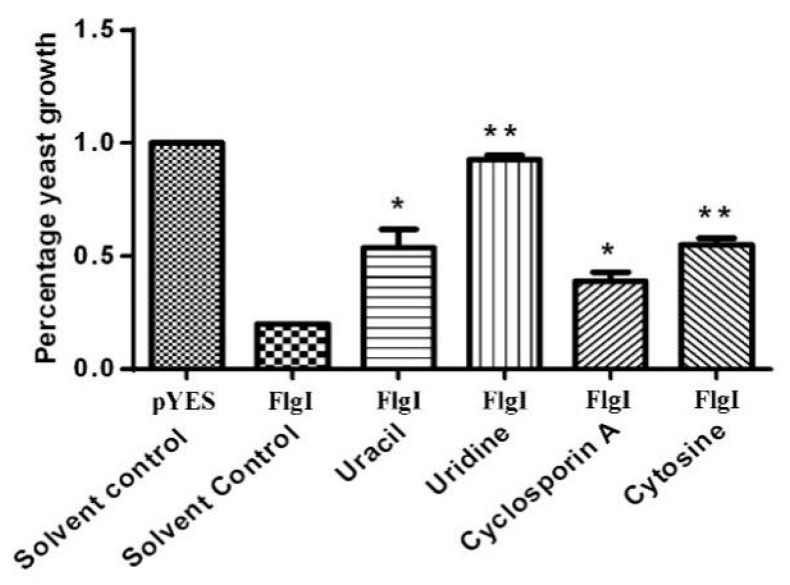
Identification of small molecular inhibitors suppresses the growth inhibition of FlgI. A cell-based yeast phenotypic assay was used to identify small molecule inhibitors that can suppress the growth inhibition of yeast cells caused by the heterologous expression of FlgI. The effect of the compounds was compared to the yeast growth in the absence of a compound (as a control for inhibition) and to the cells dividing in the absence of the effector (as a control for growth). The experiments were performed in 3 independent replicates. Bars represent the mean ± standard error, and asterisks signs indicate statistically significant differences from the control as calculated using Student’s *t*-test (** *p* < 0.01 and * *p* < 0.05).

Because FlgI is related to the inhibition of yeast cell growth and the induction of cell necrosis in tobacco plants based on the above data, we next investigated whether it is possible to identify potential inhibitors of FlgI using a large-scale screening of drug libraries in a yeast cell-based system. A previous report showed that ExoS-mediated yeast growth inhibition in *P. aeruginosa* has been successfully used to screen compounds that inhibit its activity and restore yeast growth [36]. Following a similar strategy, we performed a large-scale compound library screen to identify drugs that restore FlgI-mediated growth inhibition. In total, 1663 compounds, natural products, or, primarily, synthetic small molecules from APExBIO, Topscience, and Zining Cui’s Laboratory, were tested against FlgI. Four effective compounds were screened out, including cyclosporin A, cytosine, uracil, and uridine. Uridine was the most effective compound among them (Figure 9). A possible artifact of the inhibitor screen is the disruption of the galactose induction system. To this end, we repeated the above experiment with the CLas4425 and pYES3 strains and found that the CLas4425 strain was affected by pyrimidines but was not affected by cyclosporin A (Figure S1), which indicates that the galactose induction system is not affected by cyclosporin A and strengthens the notion that cyclosporin A directly interferes with FlgI-mediated growth inhibition. As to the effect of cyclosporin A on yeast growth, we repeated the experiment in the absence of FlgI and found that cyclosporin A alone did not affect yeast growth (Figure S2).

## 3. Discussion

Many bacterial pathogens secret proteins used for promoting infection and causing disease [11,50,51]. CLas contains a type I secretion system and the general secretory pathway responsible for exporting proteins to the periplasm; however, it lacks other secretion systems [9,15,52]. The Sec machinery promotes SDEs across the cytoplasmic membrane and is essential for bacterial survival [53]. However, how CLas translocates proteins from the periplasm to outside the outer membrane is unclear. Interestingly, OMVs were observed on the surface of *Ca*. L. solanacearum [54] and *L. crescens* [39], and it has been shown that OMVs secrete various virulence factors in other pathogens [40]. In this study, we have demonstrated that the closely related bacterium *S. meliloti* forms OMVs and present the first experimental evidence that the carboxyl-terminal, HA-tagged FlgI was secreted via OMVs in *S. meliloti*, indicating that CLas may use OMVs to secrete SDEs and other putative VFs during interactions with plant hosts.

Knowing the function of effectors in host plant cells is key to understanding the molecular mechanisms of pathogen–plant interactions and formulating sustainable management strategies. One Las-secreted effector, SDE1, has been identified as the cause of the infiltrated *N. benthamiana* leaf chlorosis or cell death [8,20] and has been shown to suppress immune responses in plants by attenuating the activity of citrus cysteine proteases [16]. SDE15 (CLIBASIA_04025) suppresses plant immunity response and promotes CLas growth in planta by targeting the citrus-susceptible gene *CsACD2* [22]. The effector CLas4425 promotes CLas proliferation by interfering with SA-mediated plant immunity [24]. Two nonclassical secreted proteins, SC2_gp095 and LasBCP (CLIBASIA_RS00445), have been identified as functional peroxidase enzymes that significantly downregulate the transcription of *RbohB*, the gatekeeper of hydrogen peroxide-mediated defense signals in plants [55,56], indicating it plays a role in secreting proteins that suppress the host innate immunity. In this study, FlgI is speculated to activate *RbohB* and *WIPK* through PAMP-triggered immunity or effector-triggered immunity in *N. benthamiana,* leading to redox imbalance, ROS production, and, eventually, cell death. However, transient FlgI-induced tobacco cell death was weaker than SDE1-induced cell death (Pitino et al., 2016) because cell necrosis in the inoculation region was not observed until 7 dpi. The underlying molecular mechanism of FlgI in the process of pathogen–plant interaction remains to be unveiled, and identifying specific target host proteins of FlgI will help to address the question of how FlgI induces plant cell death.

In order to determine the functional characteristics of pathogen effectors and how these proteins manipulate host cells, it is crucial to identify the host compartment in which these effectors are located [57]. Therefore, when evaluating effector functions, subcellular localization is one of the first considerations [8]. To investigate the subcellular localization of FlgI in plant cells, FlgI-GFP was constructed by fusing the coding sequence of *flgI* with *GFP*, and the resulting plasmid was delivered into the leaves of *N. benthamiana*. Under a Nikon A1 confocal microscope, we observed that the green fluorescence of FlgI-GFP was located in the cytoplasm, cell periphery, and nuclear periphery of tobacco cells, and there was a strong spot fluorescence signal along the cell periphery. This is consistent with the previous reports that FlgI was expressed on the cell surface of CLas and associated with the basal portion of the flagellar structure [58], and the P ring of the bacterial flagellar motor consists of multiple copies of FlgI and is located in the peptidoglycan layer (Hizukuri et al., 2006). In addition, although there is an almost complete set of genes involved in the flagellar synthesis pathway in the sequenced CLas genome [9], intact flagella has not yet been observed on the surface of CLas cells in citrus [59], indicating that some flagellar proteins may have other functions. Collectively, this indicates that FlgI may regulate host membrane system-mediated substance transport and signaling.

CLas infection perturbs normal carbon partitioning [60] and is usually accompanied by callose deposition in sieve pores [8,43]. The phloem plugging was mainly due to the accumulation of starch, which, eventually, resulted in the chlorosis of the leaf in infected *N. benthamiana* [8]. In CLas-infected citrus, genes that have been proven to participate in callose deposition were induced [60,61]. In this study, we found that FlgI induced callose deposition in the infiltrating zone 3 dpi and increased callose accumulation in cellular and vascular tissue in a manner similar to CLas-infected citrus plants [60]. This suggests that FlgI may be a protein involved in callose deposition in CLas-affected citrus. Further identification of the functions of CLas effectors associated with pathogenicity and virulence may help identify potential targets for controlling CLas infection and HLB progression.

*S. cerevisiae* has emerged as a model system for the identification and characterization of bacterial effector functions [27,28,29]. Effector proteins from plant pathogens often cause growth inhibition when expressed in yeast, such as *P. syringae* HopAA1-1, HopAD1, HopU1 [31], HopX1, HopG1, and AvrPtoB [32]; *Xanthomonas campestris* XopB, XopE1, and AvrRxo1 [29]. In addition, it has been found that the expression of translocated *Shigella* proteins resulted in more frequent and more severe yeast growth inhibition than the non-translocated proteins [28,62,63]. In our study, 15 VFs were predicted using the DeepVF and PHI-base searches from the proteome of CLas. Through heterologous expression in *S. cerevisiae*, one of them, FlgI, was found to be able to inhibit yeast growth. In addition, yeast cell-based assays are well suited for screening small molecule inhibitors that inhibit overexpressed proteins [36,37,38]. To this end, four small molecule inhibitors—cyclosporin A, cytosine, uracil, and uridine—were identified to suppress the growth inhibitory effect of the exogenous expression of FlgI. Taking the CLas4425 strain as a positive control, we excluded the possible artifact of the inhibitor screen, such as the pyrimidines, which may disrupt the galactose induction system and strengthen cyclosporin A, directly interfering with FlgI-mediated growth inhibition. It would be interesting to evaluate the antimicrobial effect of cyclosporin A on CLas in a greenhouse environment trial.

Overall, our data suggest that the virulence factor FlgI inhibits yeast cell proliferation when expressed in *S. cerevisiae*. We provide experimental evidence that FlgI is a novel Sec-dependent secretory protein and can be secreted via OMVs in *S. meliloti*. FlgI is mainly localized in the cytoplasm, cell periphery, and nuclear periphery of tobacco cells and has a strong ability to induce callose deposition and cell necrosis in *N. benthamiana.* By screening a large library of compounds in a high-throughput format, we found that cyclosporin A restored the growth of FlgI-expressing yeast. The knowledge gained from this Sec-dependent effector improves our understanding of CLas pathogenicity and contributes to the development of new and more effective strategies for the management of HLB.

## 4. Experimental Procedures

### 4.1. Bacterial and Yeast Strains

*S. cerevisiae* W303-1A (*MATa ade2-1 trp1-1 can1-100 leu2-3,112 his3-11,15 ura3-1*) was propagated on yeast–peptone–dextrose or synthetic dextrose minimal medium missing the appropriate amino acid at 28 °C or 30 °C. *E. coli* DH5α and BL21 were propagated in Luria–Bertani (LB) broth (10 g/L tryptone, 5 g/L yeast extract, and 10 g/L NaCl, pH 7.0) at 37 °C. *A. tumefaciens* GV3101 was grown in LB medium with 50 μg/mL rifampicin (Rif) at 28 °C.

### 4.2. Plasmid Constructions for the Yeast Cell-Based Assay

All CLas effectors were amplified with the Q5 High-Fidelity DNA Polymerase (Gene Biotechnology International Trade Co., Ltd., Shanghai, China) from *Citrus sinensis* genomic DNA. Oligonucleotides used for the amplification of the putative effectors are listed in Supplementary Table S2. The effector fragments were inserted in the frame into the multiple cloning sites of the pYES3 (Shanghai Yubo Biological Technology Co., Ltd., Shanghai, China). The correct integration of the genes in pYES3 was investigated using PCR with forward primer GAL1-F (5′-AATATACCTCTATACTTTAACGTC-3′) and reverse primer CYC1-R (5′-GCGTGAATGTAAGCGTGAC-3′).

### 4.3. Bioinformatic Selection of CLas Pathogenic Factor Candidates

The predicted proteome of CLas was obtained from the KEGG database (https://www.kegg.jp/kegg-bin/show_organism?org=T00948 (accessed on 21 April 2017)). The CLas proteome was submitted to the DeepVF webserver (https://deepvf.erc.monash.edu/) for VF prediction. In addition, the CLas protein sequences were searched against the PHI-base database version 4.3 (obtained from http://www.phi-base.org/), using BLASTP with an e-value ≤10^−10^ as the cutoff.

### 4.4. Yeast Growth Inhibition Assays

The expression vector pYES3/CT carrying CLas effectors was transformed into the *S. cerevisiae* strain W303-1A as previously described [64]. Transformed yeasts were plated onto selective synthetic complete–Trp media with 2% glucose. Yeast growth inhibition assays were conducted as previously described with minor modifications [29]. In brief, yeast cultures were grown overnight in liquid-selective media, washed, and normalized to an optical density at 600 nm (OD_600_) of 1.0. The cell suspension was 10-fold serially diluted 6 times and spotted (10 µL) onto repressing (glucose–Trp) and inducing (galactose + raffinose–Trp) solid-selective media. The dishes were cultured at 30 °C, and the yeast growth defects were monitored after 2 to 3 days. Growth was compared with the suitability of yeast containing the toxic pYES3-ExoY and the empty vector [36,37,38].

For yeast viability plating assays, strains containing pYES3/CT-based expression vectors carrying CLas effectors were grown in selective media. The culture was diluted to an OD_600_ of 0.5, recovered in a fresh repressing media for 2 h, and then normalized to an OD_600_ of 0.2 and induced in a medium containing galactose (2%) and raffinose (1%). At different time points after induction, the same amount was collected, with 10-fold being serially diluted 5 times, and 10 µL was speckled onto the repressing solid medium. The number of colonies was counted after incubation at 30 °C for 2 days.

### 4.5. Prediction and Confirmation of FlgI Signal Peptide

The Sec-secretion SP of FlgI was predicted using SignalP 5.0 [65], Phobius, and SPOCTOPUS [66]. An *E. coli phoA* gene (GenBank No. NC_000913) fusion assay [15,17] was used to validate the putative SP of FlgI. The *phoA* gene without its native SP-encoding sequence (m*phoA*) was amplified from *E. coli* with the primers of mphoA-F and phoA-R (Supplementary Table S2) and then fused with the *Nde*I/*Xho*I double-digested pET-28a (+) using a ClonExpress Ultra One Step Cloning Kit (Vazyme Biotech Co., Ltd., Nanjing, China), resulting in pET-mphoA. pET-phoA containing the full-length *phoA* gene was used as a positive control. The DNA fragment of *flgI* containing SP (N-terminal 20 amino acids) was amplified from the CLas genome with primers FlgI SP-F/FlgI SP-R. Then, the second round of PCR was performed using the PCR products of FlgI SP and *mphoA* with primers FlgI SP-F/phoA-R. The product of the second round PCR was ligated into pET-mphoA through homologous recombination to obtain the pET-FlgISP-mphoA. The sequences of plasmids were confirmed using DNA sequencing; then, plasmids were transformed into the component *E. coli* BL21 cells. The PhoA activity of the transformants was detected on LB plates containing 90 μg/mL BCIP that used chromogenic PhoA as a substrate, as well as 100 mM isopropyl β-D-1-thiogalactopyranoside (IPTG), which induced the *lacUV5* promoter and *T*7*lac* promoter, and 75 mM Na_2_HPO_4_, which blocked endogenous phosphatase activity. *E. coli* BL21 cells carrying pET-phoA were used as a positive control, and BL21 cells carrying pET-mphoA were used as a negative control. After incubation at 37 °C for 16h, the color of the transformants changed to blue, indicating that they had PhoA activity, while colonies that remained white indicated that they lacked PhoA activity.

### 4.6. Isolation of Outer Membrane Vesicles and Analysis using Nanoparticle Tracking and SDS-PAGE Gel Electrophoresis

The carboxyl-terminal, HA-tagged (hemagglutinin, YPYDVPDYA) FlgI expression vector was constructed using the pBBR1MCS-5 vector and transformed into the *S. meliloti* 1021 strain. The strain was grown in TY medium (5 g/L Trypton, 3 g/L Yeast Extract, 0.7 g/L CaCl_2_, pH 7.0) for 24. OMVs were isolated and purified from cell culture media using Amicon ultra-15 centrifugal filters (Guangzhou Angke Biotechnology Co., Ltd., Guangzhou, China) and size-exclusion chromatography as previously described [41]. Briefly, media was collected and spun at 5000× *g* for 5 min to remove floating cells. The supernatant was further centrifuged at 10,000× *g* for 30 min to remove cellular debris then filtrated through 0.45 μm and 0.2 μm filters (Guangzhou Angke Biotechnology Co., Ltd., Guangzhou, China) to remove larger microvesicles. The supernatant was concentrated with Amicon ultra-15 centrifugal filter units with an ultravel-100 membrane. This step not only concentrates OMVs but also eliminates a large portion of contaminated proteins with less than 100 kDa. Concentrated material was applied onto a 50 mL column packed with Sepharose CL-2B and then equilibrated with 5 mM sodium phosphate (pH 7.0, 100 kDa filtered, buffer A). The column was eluted with buffer A and 1.8 mL fractions were collected. For each fraction, the number of particles was detected using Nanosight, and protein concentrations were measured with Nanodrop 2000 (NanoDrop 2000 Technologies, Wilmington, NC, USA) or Pierce 660 nm protein assays (Shanghai Yubo Biological Technology Co., Ltd., Shanghai, China). This step of size-exclusion chromatography effectively removed contaminating proteins from OMV preparations. The size and concentration of the purified EVs were analyzed using a NanoSight LM10 instrument. Temperature was set to 25 °C. Capture screen gain was set to 1.3, and the camera level was set to 13. Process detection was threshold set to 5. Videos recorded were analyzed with Nanoparticle Tracking Analysis software version 3.0. Purified OMVs were boiled in SDS-PAGE sample loading buffer and then run on a 10% Bis-Tris gel and stained with InstantBlue (Sigma–Aldrich, Taufkirchen, Germany). At the same time, the precipitated *S. meliloti* cells were harvested and ultrasonic lysed with ice pre-cold cell lysis buffer (50 mM NaH_2_PO_4_, 300 mM NaCl, 10 mM imidazole, and 1 mM PMSF pH 8.0). Cell lysates were collected and mixed with the SDS-PAGE sample loading buffer. Mixed samples were boiled and subjected to SDS-PAGE gel and electroblotted onto a polyvinylidene difluoride membrane (Amersham Biosciences, Amersham, UK). The membranes were blotted with the HA antibody (Vazyme Biotech Co., Ltd., Nanjing, China) (1:2000 dilutions) and then the peroxidase-conjugated AffiniPure Goat Anti-Rabbit IgG (AmyJet Scientific Inc, Wuhan, China) (1:50,000 dilutions). Signals were detected with Clarity TM Western ECL Substrate (Bio-Rad, Hercules, CA, USA), and images were captured using an Analytik-jena imaging system (Analytik-jena, Jena, Germany).

### 4.7. Agrobacterium-Mediated Transient Expression of FlgI in N. benthamiana

The *flgI* was amplified with the primers FlgI-GFP-F and FlgI-GFP-R (Supplementary Table S2), using CLas strain psy62 genomic DNA as a template, then cloned into the binary vector pCAMBIA-35S-GFP to obtain pC35S-FlgI-GFP, which can express the carboxyl-terminal GFP fusion protein FlgI-GFP. This vector was then transformed into *A. tumefaciens* GV3101 using a freeze-thawing method with liquid nitrogen, and the agroinfiltration of the leaves of *N. benthamiana* was carried out as previously described with minor modifications [17,67]. Agroinfiltrated plants were kept in an incubator with 16 h of light and 8 h of dark at 25 °C. The phenotypic changes of the agrobacteria-infiltrated leaves were observed then detached from plants and visualized using GFP fluorescence under a Nikon A1 confocal microscope (Nikon, Tokyo, Japan) at 3 dpi for the protein subcellular localization analysis. The experiments were repeated at least twice.

### 4.8. Transient Expression of FlgI Induced Electrolyte Leakage and Callose Deposition in N. benthamiana

To quantify cell death, electrolyte leakage was performed as previously described (Pitino et al., 2016). Three leaf discs were collected from the pC35S-FlgI-GFP and control *Agrobacterium*-infiltrated areas at 7 dpi and immersed in 10 mL of double-distilled water and shaken at 160 rpm for 1 h; then, the conductivity of the solution was measured. Aniline blue staining allowed us to observe the presence of callose using fluorescence microscopy in *N. benthamiana* leaves agroinfiltration with pC35S-FlgI-GFP or pC35S-GFP. At 4 dpi, the callose deposition was detected with aniline staining using the method described previously [8,43], with little modification. Briefly, the leaves were cleared and dehydrated in 100% ethanol. The cleared leaves were washed in distilled water and incubated at room temperature in 0.01% aniline blue and 150 mM K_2_HPO_4_ (pH 12) for 2 h. The stained materials were mounted in 50% glycerol and observed under a Leica epifluorescence microscope (Leica Microsystems Shanghai Ltd., Shanghai, China). The number of foci of callose deposition was counted per mm^2^ for each leaf. The experiments were performed using triplicates from 3 different plants.

### 4.9. RNA Isolation and RT-qPCR Analysis

*N. benthamiana* leaves were agroinfiltrated with the pC35S-FlgI-GFP or the pC35S-GFP control. The infiltrated zones were harvested 2 dpi for RNA isolation using Easystep Super Total RNA Extraction Kit (Shanghai Promega Biological Products Ltd., Shanghai, China). Total RNAs were quantified using the Nanodrop ND-1000 spectrophotometer (NanoDrop 2000 Technologies, Wilmington, NC, USA). The cDNA was synthesized using a HiScript Ⅱ Q RT SuperMix for qPCR kit (Vazyme Biotech Co., Ltd., Nanjing, China). qPCR was performed on a QuantStudioTM 6 Flex System (Applied Biosystems, Singapore). The 20 μL reaction system contained 10 μL 2 × ChamQ Universal SYBR qPCR Master Mix (Vazyme Biotech Co., Ltd., Nanjing, China), 2.5 μmol/L of each gene-specific primers (Supplementary Table S3), and 2 μL of the cDNA sample. The following program was used: 95 °C for 30 s, 40 cycles of 95 °C for 10 s, and 60 °C for 30 s. Primers of NbEF1α (an internal control) and NbFLS2 were used as previously reported [43], and NbWIPK and NbRbohB were used as in Segonzac’s and Yoshioka’s study [47,48]. NbPlastocyanin was used as in Chakravarthy’s report [49], and NbBAK1 and NbSGT1 were used as in Zou’s work [46]. The expression level was normalized against the internal control NbEF1α. All data were statistically analyzed using the Student’s *t*-test.

### 4.10. Screening of Small Molecular Inhibitors of FlgI

In total, 550 compounds from DiscoveryProbe™ Natural Product Library (APExBIO), 1058 compounds from Natural Compound Library for HTS (Topscience), and 55 compounds from Zining Cui’s Laboratory (South China Agricultural University, Guangzhou, China) were screened at a final concentration of 33.3 μmol/L. The *S. cerevisiae* strain W303-1A, harboring the plasmid pYES-FlgI, was grown overnight in liquid-selective media containing 2% glucose. Then, the yeast cells were collected and suspended in inducing media containing galactose (2%) and raffinose (1%) to a cell density OD_600_ of 1.0. The cultures were aliquoted into wells of 96-well plates, and then compounds and the solvent control DMSO were added. Plates were cultured at 30 °C, 250× *g*, and the growth recovery of yeast was examined after 48 h. As a control, cells containing empty vector pYES3 /CT were also grown, diluted, and inoculated with the solvent DMSO. The effect of the hits on yeast growth recovery was quantified in the percentage of growth as previously described [36].

## Figures and Tables

**Table 1:** Features of 15 CLas putative effector proteins.

Genebank Accession	Gene ID	Sequence Length (aa) ^a^	Annotation ^b^
*ACT56745*	CLIBASIA_00785	216	ATP-dependent Clp protease proteolytic subunit
*ACT56849*	CLIBASIA_01305	369	flagellar basal body P-ring protein FlgI
*ACT56851*	CLIBASIA_01315	262	flagellar basal body rod protein FlgG
*ACT56968*	CLIBASIA_01910	205	superoxide dismutase
*ACT57006*	CLIBASIA_02100	423	3-oxoacyl-(acyl carrier protein) synthase II
*ACT57010*	CLIBASIA_02120	294	periplasmic solute binding protein
*ACT57011*	CLIBASIA_02125	280	ABC transporter, nucleotide binding/ATPase protein (iron)
*ACT57012*	CLIBASIA_02130	273	ABC transporter, membrane spanning protein (iron)
*ACT57013*	CLIBASIA_02135	277	ABC transporter, membrane spanning protein (iron transport)
*ACT57309*	CLIBASIA_03655	674	putative type I restriction-modification system DNA methylase
*ACT57310*	CLIBASIA_03660	426	putative restriction endonuclease S subunit
*ACT57322*	CLIBASIA_03720	551	chaperonin GroEL
*ACT57350*	CLIBASIA_03870	853	ATP-dependent Clp protease
*ACT57580*	CLIBASIA_05045	542	phosphoglucomutase
*ACT57591*	CLIBASIA_05100	502	lysyl-tRNA synthetase

aa ^a^: amino acid; annotation ^b^ was derived from CLas genome sequence in NCBI.

## Data Availability

Data are contained within the article and supplementary materials.

## Supplementary Materials

The following supporting information can be downloaded at: https://www.mdpi.com/article/10.3390/plants13020318/s1.
