# Supplementary material for: FlgI Is a Sec-Dependent Effector of Candidatus Liberibacter asiaticus That Can Be Blocked by Small Molecules Identified Using a Yeast Screen
Source: Plants (Basel). 2024 Jan 21;13(2):318. doi: 10.3390/plants13020318 (PMC10819201; doi:10.3390/plants13020318)
Supplement: Supplementary file 1 [file plants-13-00318-s001.zip › plants-2783612-Supplementary figures-main.pdf]

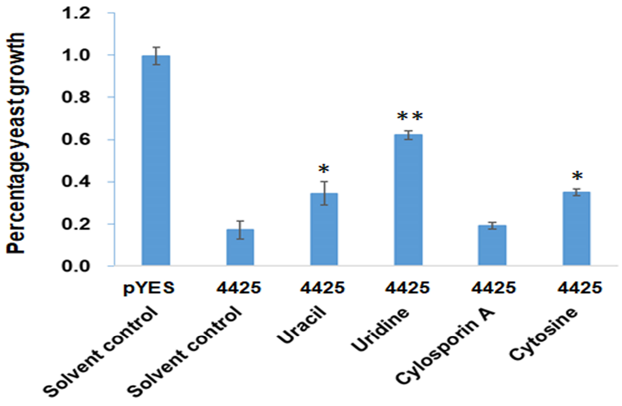

Figure S1

Figure S1. Identification of small molecular inhibitors suppresses the growth inhibition of Clas4425. A cell-based yeast phenotypic assay was used to identify small molecule inhibitors which can suppress the growth inhibition of yeast cells caused by heterologous expression of Clas4425. Effect of the compounds was compared to the yeast growth in absence of compound (as control for inhibition) and to the cells dividing in absence of the effector (as a control for growth). The experiments were performed in 3 independent replicates. Bars represent the mean ± standard error, and asterisks signs indicate statistically significant differences from the control as calculated using Student's *t*-test (**p<0.01 and *p<0.05).

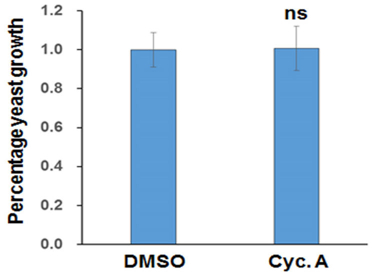

Figure S2

Figure S2. The effect of cyclosporin A alone on yeast growth. Cell-based yeast phenotypic analysis was used to determine the effect of cyclosporin A on yeast growth in the absence of FlgI. Effect of the compound was compared to the yeast growth in the solvent control DMSO. The experiments were performed in 3 independent replicates. Bars represent the mean ± standard error. The percentage of yeast growth was compared using Student's t-test. (ns: not significant, n = 3).
