# Supplementary material for: FlgI Is a Sec-Dependent Effector of Candidatus Liberibacter asiaticus That Can Be Blocked by Small Molecules Identified Using a Yeast Screen
Source: Plants (Basel). 2024 Jan 21;13(2):318. doi: 10.3390/plants13020318 (PMC10819201; doi:10.3390/plants13020318)
Supplement: Supplementary file 1 [file plants-13-00318-s001.zip › Supplementary Table 2. Sequences of oligonucleotides for amplification putative effectors.pdf]

Supplementary Table 2. Sequences of oligonucleotides used for amplification putative effectors

Primers	Sequences of oligonucleotides（5'-3’)	Restriction site
HLB1910-F	CGCGGATCCATGGACTTTAAACTGCCAAATTTACCTTATAG	BamHI
HLB1910-R	CCGGAATTCAGTCACCTTTTGAGCTAGCATAGCTGTC	EcoRI
HLB2100-F	CTTGGTACCGAGCTCGGATCCATGAGACGTGTGGTTGTAACGGGAC	BamHI
HLB2100-R	TGCTGGATATCTGCAGAATTCGATATTATCTTGAAACTTTCTAAATAC	EcoRI
HLB2120-F	CGCGGATCCATGTTACGTTATTTTATATGTTTGTTGTTC	BamHI
HLB2120-R	CCGGAATTCAAATAAAGTATCCACAATTTTCGTTAGAGAG	EcoRI
HLB2125-F	CGCGGATCCATGAAAACAGCTGTCGCTTCCACGAAAGGTGG	BamHI
HLB2125-R	CCGGAATTCGCTTTTTTTTATGCACATGGTGGAATTTATTTG	EcoRI
HLB2130-F	CGCGGATCCATGATAAATCAAATTTTAGAACCTTTTACATAC	BamHI
HLB2130-R	CCGGAATTCTGAGGTTCTGTATTGTTTAGAGAAAAAGAAAAG	EcoRI
HLB2135-F	CGCGGATCCATGAGTAGTTTCCTACATTATGTTCTGTTGCC	BamHI
HLB2135-R	CCGGAATTCGGCAGTTCTTTTATTGATAATTATTGAATATGC	EcoRI
HLB3655-F	CTTGGTACCGAGCTCGGATCCATGACTGAGTTCACAGGTTCTGCGGCGTCG	BamHI
HLB3655-R	TGCTGGATATCTGCAGAATTCCTCTGTCGCCATCTCTTCCAATAAGGTTGC	EcoRI
HLB3660-F	CGCGGATCCGTGAAGCACTATAAAGCTTACCCACAAT	BamHI
HLB3660-R	CCGGAATTCTTGCGACTCTCCTCGCAAGTCAATCTGG	EcoRI
HLB3720-F	CGCGGATCCATGGCTGCTAAAGATATTAAGTTGGGTACTGC	BamHI
HLB3720-R	CCGGAATTCCATCATCATATCCATACCACCCATACCAC	EcoRI
HLB3870-F	CGCGGATCCATGAATAGTGATAAATATTCAGACTTGATG	BamHI
HLB3870-R	CCGGAATTCAATAACTCGAAAATTTAGGTTATCATCATCAAC	EcoRI
HLB5045-F	CTTGGTACCGAGCTCGGATCCATGTTGTGCACTATAGTCCCCACTGTTCCTTATC	BamHI
HLB5045-R	TGCTGGATATCTGCAGAATTCTGAGGCAATCGAGGGATTTGTATGTCCTATAT	EcoRI
HLB5100-F	CGCGGATCCATGAATAATACTCTTACTCCCGAATC	BamHI
HLB5100-R	CCGGAATTCTTCTGTTTTCCGACTACCATTTTTCACACG	EcoRI
HLB0785-F	CGCGGATCCATGAGTTTTGAGGGATGTGCAAAAAAGGATTCC	BamHI
HLB0785-R	CCGGAATTCTTTGCTCTTTTCTTCAATATCAATTCGAGAC	EcoRI
HLB1305-F	CTTGGTACCGAGCTCGGATCCATGATTTTTCGTATAGCTTTTTTGATTTTTTC	BamHI
HLB1305-R	TGCTGGATATCTGCAGAATTCTTGAATTATAATCTCTGATTGAAGGGCGCCCG	EcoRI
HLB1315-F	CTTGGTACCGAGCTCGGATCCATGAAAGCATTTATGATCGCAGCAGC	BamHI
HLB1315-R	TGCTGGATATCTGCAGAATTCTCTCATCTTAGTAATTAAAGAAGACATCTC	EcoRI
CYC1-R	GCGTGAATGTAAGCGTGAC	NA
GAL1-F	AATATACCTCTATACTTTAACGTC	NA
mphoA-F	5′-TGCCGCGCGGCAGCCATATGCCAGAAATGCCTGTTCTGGAA-3′	NdeI
phoA-R	5′-TGGTGGTGGTGGTGCTCGAGTTATTTCAGCCCCAGAGCGG -3′	Xho I
flgISP-F	5′-TGCCGCGCGGCAGCCATATGATTTTTCGTATAGCTTT TTT-3′	NdeI
flgISP-R	5′-TTCCAGAACAGGCATTTCTGGTGCTGATATCTCA TGCAA-3′	NA
flgI-GFP-F	5′-TGCTCTAGAATGATTTTTCGTATAGCTTTTTTGATTTTTTC-3′	XbaI
flgI-GFP-R	5′-CGCGGATCCTTGAATTATAATCTCTGATTGAAGGGCGCCCG-3′	BamHI
HLB4425-F	CGCGGATCCATGAAGAAGTATATCACATTATTAAC	BamHI
HLB4425-R	CCGGAATTCCTGGGATCGGTAGTTTCGATAATATCC	EcoRI
