# Supplementary material for: FlgI Is a Sec-Dependent Effector of Candidatus Liberibacter asiaticus That Can Be Blocked by Small Molecules Identified Using a Yeast Screen
Source: Plants (Basel). 2024 Jan 21;13(2):318. doi: 10.3390/plants13020318 (PMC10819201; doi:10.3390/plants13020318)
Supplement: Supplementary file 1 [file plants-13-00318-s001.zip › Supplementary Table 3. Sequences of oligonucleotides for RT-qPCR.pdf]

Supplementary Table 3. Sequences of oligonucleotides used for RT-qPCR

**Primers**	**Sequences of oligonucleotides（5'-3′)**
RbohB-F:	TTTCTCTGAGGTTTGCCAGCCACCACCTAA
RbohB-R:	GCCTTCATGTTGTTGACAATGTCTTTAACA
WIPK-F:	CCGATCTGCCCCGTTCCATCC
WIPK-R:	TCAGGATTCAGCGACAAAGCTTCC
Plasto-F:	ATCCCATACAAGACGCTAAAACAA
Plasto-R:	GCTAACCCGCCTACACG
BAK1-F	TGAACGGTTGCTTGTTTATCCATAT
BAK1-R	CAGAGAAGAGCTACCCGAATAAG
SGT1-F	CAGAGGAGGTGGTGGTGACTATA
SGT1-R	GGAGGGCTTCCTTCGACCTTCTTTG
EF1a-F	TTGCCTTGTGGAAGTTTGAGAC
EF1a-R	CATACCAGGCTTGAGGACACCAGTT
FLS2-F	TCAAATGGTGGATGACTGGA
FLS2-R	ATGATATGCTGCTCCCATCC
